# Supplementary material for: Examining the Role of Anxiety and Depression in Dietary Choices among College Students
Source: Nutrients. 2020 Jul 11;12(7):2061. doi: 10.3390/nu12072061 (PMC7400947; doi:10.3390/nu12072061)
Supplement: Supplementary file 1 [file nutrients-12-02061-s001.pdf]

Table S1. HEI components and scoring standards

Components	Maximum points	Standard for maximum score	Standard for minimum score of zero
Total fruits	5	≥0.8 cup equivalent per 1,000 kcal	No fruit
Whole fruits	5	≥0.4 cup equivalent per 1,000 kcal	No whole fruit
Total vegetables	5	≥1.1 cup equivalent per 1,000 kcal	No vegetables
Greens and beans	5	≥0.2 cup equivalent per 1,000 kcal	No dark green vegetables or legumes
Whole grains	10	≥1.5 cup equivalent per 1,000 kcal	No whole grains
Dairy	10	≥1.3 cup equivalent per 1,000 kcal	No dairy
Total protein foods	5	≥2.5 cup equivalent per 1,000 kcal	No protein foods
Seafood and plant proteins	5	≥0.8 cup equivalent per 1,000 kcal	No seafood or plant proteins
Fatty acids	10	(PUFAs [a] + MUFAs [b])/SFAs [c] ≥2.5	(PUFAs [a] + MUFAs [b]) /SFAs [c] ≤1.2
Refined grains	10	≤1.8 oz equivalent per 1,000 kcal	≥4.3 oz equivalent per 1,000 kcal
Sodium	10	≤1.1 gram per 1,000 kcal	≥2.0 grams per 1,000 kcal
Added sugars	10	≤6.5% of energy	≥26% of energy
Saturated fats	10	≤8% of energy	≥16% of energy

Note. Reprinted from National Cancer Institute [50]. Retrieved from https://epi.grants.cancer.gov/hei/developing.html.

[a] PUFA=Polyunsaturated fatty acid. [b] MUFA=Monounsaturated fatty acid. [c] SFA=Saturated fatty acid.

Table S2. Descriptive statistics of main study variables and BMI (N=225)

	Mean	SD	Skewness(SE)	Kurtosis(SE)
Age	18.91	1.467	2.123(.163)	4.51(.32)
GAD-7 score	5.15	5.09	1.32(.16)	1.49(.32)
PHQ-9 score	5.88	4.90	1.16(.16)	1.20(.32)
Whole grain [a]	2.19	3.33	1.39(.16)	0.52(.32)
Dairy [a]	5.18	3.65	-0.04(.16)	-1.46(.32)
Fatty acid [a]	5.20	3.90	0.01(.16)	-1.56(.32)
Sodium [a]	3.61	3.39	0.47(.16)	-1.10(.32)
Refined grain [a]	4.95	3.86	0.01(.16)	-1.54(.32)
Saturated fat [a]	5.70	3.61	-0.26(.16)	-1.32(.32)
Sugar [a]	7.16	3.15	-0.88(.16)	-0.48(.32)
Fruit [a]	3.45	4.13	0.67(.16)	-1.30(.32)
Vegetable [a]	4.26	3.48	0.45(.16)	-1.19(.32)
Protein [a]	6.03	3.11	-0.15(.16)	-0.97(.32)
HEI total score	47.73	14.70	0.51(.16)	-0.28(.32)
Total caloric intake	1827.40	838.43	0.85(.16)	0.85(.32)
BMI	23.55	4.61	1.42 (.16)	2.57 (.32)

[a] HEI component scores

Table S3. Correlation of main study variables and BMI

	1	2	3	4	5	6	7	8	9	10	11	12	13	14
1	1													
2	0.80	1												
3	-0.00	-0.03	1											
4	-0.02	-0.02	0.05	1										
5	-0.03	-0.00	0.18	-0.50	1									
6	0.11	0.16	0.10	-0.08	0.04	1								
7	0.09	0.09	0.35	-0.01	0.06	0.24	1							
8	-0.00	0.02	0.18	-0.48	0.63	0.09	-0.91	1						
9	-0.21	-0.23	0.11	0.11	0.15	-0.42	-0.01	-0.06	1					
10	-0.01	-0.02	0.14	-0.06	0.21	0.23	0.18	0.28	0.06	1				
11	-0.05	-0.11	0.10	0.08	0.08	-0.13	0.14	0.01	0.26	0.15	1			
12	-0.07	-0.03	0.18	0.03	0.06	-0.02	0.27	0.02	0.12	0.08	0.29	1		
13	-0.04	-0.03	0.57	0.02	0.49	0.27	0.53	0.40	0.29	0.59	0.47	0.47	1	
14	-0.23	-0.20	-0.09	0.13	-0.14	0.18	-0.01	-0.21	0.00	0.03	-0.10	0.16	-0.02	1

Note. 1=GAD-7 score. 2=PHQ-9 score. 3=Whole grain. 4=Dairy. 5=Fatty acid. 6=Sodium. 7=Refined grain. 8=Saturated fat. 9=Sugar. 10=Fruit. 11=Vegetable. 12=Protein. 13=HEI total score. 14=Total caloric intake.

*$p<.05$. **$p\leq.01$. ***$p\leq.001$

Table S4. The effects of depression on food choices across sex

HEI component scores	*b*(SE)	*p*-value
**Female**		
Whole grain	-.04(.05)	.50
Dairy	.01(.05)	.92
Fatty acid	-.02(.06)	.78
Sodium	.07(.05)	.17
Refined grain	.07(.06)	.21
Saturated fat [a]	.07(.05)	.19
Sugar	-.17(.05)	.00***
Fruit [a]	.00(.07)	1.00
Vegetable [a]	-.04(.06)	.53
Protein	-.01(.04)	.81
**Male**		
Whole grain	-.04(.05)	.50
Dairy	.01(.05)	.92
Fatty acid	-.02(.06)	.78
Sodium	.07(.05)	.17
Refined grain	.07(.06)	.21
Saturated fat [a]	-.15(.08)	.04*
Sugar	-.17(.05)	.00***
Fruit [a]	-.19(.08)	.01**
Vegetable [a]	-.25(.07)	.00***
Protein	-.01(.04)	.81

[a] Paths with significant sex differences

*p<.05. **p≤.01. ***p≤.001.

Table S5. Comparison of USDA recommendation with the potential effects of severe anxiety and depression on sugar and saturated fat intake

	HEI score	Contribution to energy (%)
Sugar		
USDA recommendation	10	≤6.5
Severe anxiety	3.36	+6.6
Severe depression	4.59	+9
Saturated fat		
USDA recommendation	10	≤8%
Severe depression	4.05	+3.24%

Table S6. Comparison of USDA recommendation with the potential effects of severe depression on fruit and vegetable intake

	HEI score	Total fruits consumed (cups per 1000 cal)	Whole fruits consumed (cups per 1000 cal)
Fruits			
USDA recommendation	10	≥.8	≥.4
Max depression	-5.13	-.41	-.21
Vegetables		Total vegetables consumed (cups per 1000 cal)	Greens and beans consumed (cups per 1000 cal)
USDA recommendation	10	≥1.1	≥.2
Max depression	-6.75	-.74	-.14
